# Supplementary material for: Risk factors for excess all-cause mortality during the first wave of the COVID-19 pandemic in England: A retrospective cohort study of primary care data
Source: PLoS One. 2021 Dec 9;16(12):e0260381. doi: 10.1371/journal.pone.0260381 (PMC8659693; doi:10.1371/journal.pone.0260381)
Supplement: S1 Appendix — (PDF) [file pone.0260381.s001.pdf]

S1 Appendix. Defining Usual Mortality Ratio, Excess Mortality Ratio and True Pandemic Interaction

Consider the simplest situation, a single pandemic period with a combined non-pandemic comparison or reference period, and a dichotomous risk factor. Using upper case for actual rates or rate ratios, and lower case for the corresponding parameters from log-linear modelling (we will use a Poisson model):

Log_e (mortality rate) = a + u*x_{1i} + p*x_{2i} + i*x_{3i}

where a = constant, u = usual effect of exposure, p = pandemic period, i = interaction of exposure and pandemic; x_{1i} is a dichotomous variable indicating exposure (=0 if not exposed, =1 if exposed); x_{2i} is a dichotomous variable indicating pandemic (=0 if 2015-9, =1 if 2020); x_{3i} is a dichotomous variable representing the interaction of exposure and pandemic (=1 if period=2020 and exposed, else =0).

**Period**	**Exposure**	**Modelled mortality rate**
0	0	A = exp [a]
0	1	B = exp [a + u]
1	0	C = exp [a + p]
1	1	D = exp [a + u + p + i]

The observed rates C and D are mixtures of “usual” and “excess” deaths. We want to assess whether the effect of the exposure differs between these two components, expressed as a “true interaction rate ratio”. We can estimate the relative effect of the exposure on the “usual” deaths directly (exp(e)= E), but we can only estimate the effect of the exposure on the “excess” deaths indirectly, as follows:

**Exposure**	**“Usual” Rate**	**Pandemic Rate**	**Excess Rate = Pandemic Rate – Usual Rate**
0	A	C = A*P	(A*P) – A = A*(P - 1)
1	B = A*U	D = A*U*P*I	(A*U*P*I) - (A*U) = A*U*(P*I -1)
Relative effect of 1 vs 0	U	U*I	$\frac{U*(P*I - 1)}{P - 1}$ = E

where A = usual mortality in non-exposed, U = relative effect of exposure on usual deaths, P = exp[p] = relative effect of pandemic on non-exposed deaths, I = exp[i] = interaction effect of pandemic and exposure (relative effect of pandemic on exposed vs. non-exposed *or* relative effect of exposure in pandemic vs non-pandemic periods), E = relative effect of exposure on excess deaths

The “true interaction ratio” (comparing <u>excess</u> to usual deaths) (T) is thus:

$$T = \frac{E}{U} = \frac{P * I - 1}{P - 1}$$

Which can be rewritten as:

$$E = T * U$$

On the log-scale this can be-written as:

$$\ln(E) = \ln(T) + \ln(U) = \left[\frac{\ln(T)}{\ln(I)}\right] * \ln(I) + \ln(U) = k * \ln(I) + \ln(U)$$

where k is the scalar applied to the *lincom* command in Stata to provide a 95% confidence interval for E based on the assumption that the Wald test for I and E is the same.

Note, in the text we refer to U and E as the UMR (Usual Mortality Ratio) and EMR (Excess Mortality Ratio) respectively.
