# Supplementary material for: Risk factors for excess all-cause mortality during the first wave of the COVID-19 pandemic in England: A retrospective cohort study of primary care data
Source: PLoS One. 2021 Dec 9;16(12):e0260381. doi: 10.1371/journal.pone.0260381 (PMC8659693; doi:10.1371/journal.pone.0260381)
Supplement: S1 Fig — (PDF) [file pone.0260381.s002.pdf]

S1 Figure: Weekly excess mortality during 2020 using CPRD versus ONS national figures for England

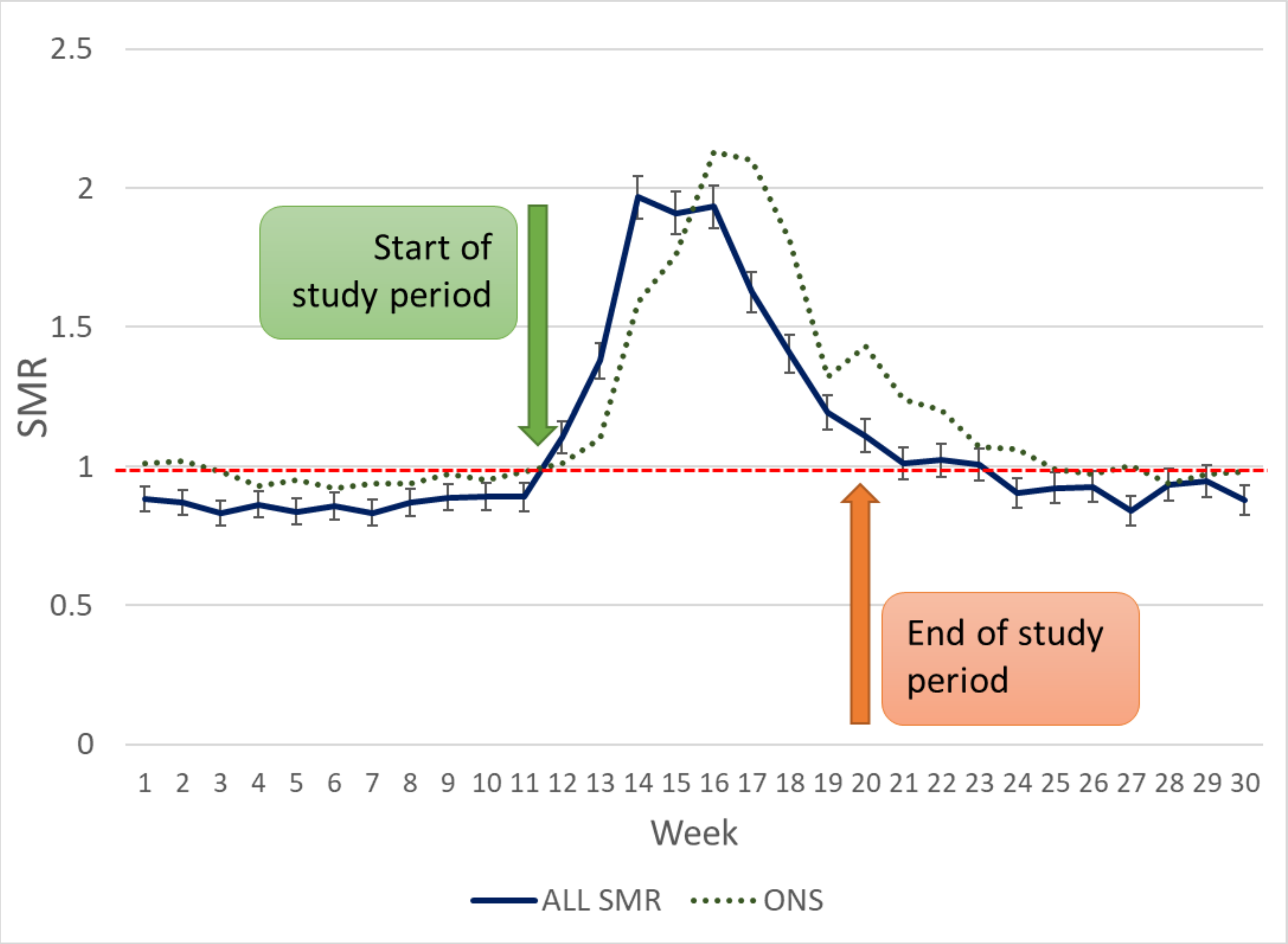

ONS – Office for National Statistics. Excess morality figures for England in 2020 available at
https://www.ons.gov.uk/peoplepopulationandcommunity/birthsdeathsandmarriages/deaths/datasets/excessdeathsinenglandandwales2020final
