# Supplementary material for: Risk factors for excess all-cause mortality during the first wave of the COVID-19 pandemic in England: A retrospective cohort study of primary care data
Source: PLoS One. 2021 Dec 9;16(12):e0260381. doi: 10.1371/journal.pone.0260381 (PMC8659693; doi:10.1371/journal.pone.0260381)
Supplement: S1 Table — (PDF) [file pone.0260381.s004.pdf]

S1 Table: Number of total patients and recorded deaths in 2020 and 2015-9

	2020 Total patients	2020 % of total	2020 Total deaths	2020 % who died	2015-9 Average total patients	2015-9 Average total deaths	2015-9 % who died
**Sex**							
- Females	2,440,573	50.47%	8,327	0.34%	2,318,138.0	5,286.4	0.23%
- Males	2,395,135	49.53%	8,408	0.35%	2,261,175.4	5,040.0	0.22%
**Age**							
- 30 to 39	1,107,502	22.90%	146	0.01%	1,031,082.8	110.6	0.01%
- 40 to 49	1,008,371	20.85%	355	0.04%	1,007,627.2	283.4	0.03%
- 50 to 59	1,026,969	21.24%	904	0.09%	957,067.0	611.0	0.06%
- 60 to 69	756,480	15.64%	1,759	0.23%	732,154.4	1,229.2	0.17%
- 70 to 79	587,255	12.14%	3,675	0.63%	525,487.4	2,266.2	0.43%
- 80 to 89	287,517	5.95%	6,190	2.15%	267,730.0	3,664.8	1.37%
- 90 to 104	61,614	1.27%	3,706	6.01%	58,164.6	2,161.2	3.72%
**Smoking**							
- Never	2,184,223	45.17%	5,772	0.26%	2,059,639.6	3,432.6	0.17%
- Ex	1,660,167	34.33%	8,557	0.52%	1,537,885.0	4,923.8	0.32%
- Current	883,863	18.28%	1,974	0.22%	861,890.4	1,549.6	0.18%
- Unknown	107,455	2.22%	432	0.40%	119,898.4	420.4	0.35%
**Ethnicity**							
- White	3,284,482	67.92%	12,173	0.37%	3,105,139.4	7,247.4	0.23%
- Black	139,459	2.88%	365	0.26%	126,407.6	123.0	0.10%
- Asian	288,974	5.98%	484	0.17%	247,025.4	194.6	0.08%
- Mixed	89,162	1.84%	180	0.20%	73,574.4	66.2	0.09%
- Other	139,479	2.88%	218	0.16%	111,446.0	83.4	0.07%
- Unknown	894,152	18.49%	3,315	0.37%	915,720.6	2,611.8	0.29%
**Deprivation**							
- IMD1 (Least)	1,140,503	23.59%	3,645	0.32%	1,104,722.4	2,289.0	0.21%
- IMD2	997,546	20.63%	3,523	0.35%	952,221.4	2,153.8	0.23%
- IMD3	958,205	19.82%	3,388	0.35%	904,390.4	2,078.0	0.23%
- IMD4	917,876	18.98%	3,138	0.34%	854,847.0	1,930.6	0.23%
- IMD5 (Most)	821,578	16.99%	3,041	0.37%	763,132.2	1,875.0	0.25%
**BMI**							
- <20 vs 20-30	250,583	5.18%	2,119	0.85%	234,603.4	1,327.4	0.57%
- 20-30	2,968,693	61.39%	9,012	0.30%	2,848,523.0	5,551.2	0.19%
- 30-35 vs 20-30	724,659	14.99%	2,037	0.28%	679,379.6	1,171.0	0.17%
- 35-40 vs 20-30	268,650	5.56%	744	0.28%	244,690.0	415.0	0.17%
- 40+ vs 20-30	147,084	3.04%	507	0.34%	129,771.6	250.0	0.19%
- Unknown	476,039	9.84%	2,316	0.49%	442,345.8	1,611.8	0.36%
**Region**							
- Non-London	3,999,810	82.71%	14,407	0.36%	3,818,522.8	9,193.4	0.24%
- London	835,898	17.29%	2,328	0.28%	760,790.6	1,133.0	0.15%
**Care Home**							
- Yes	27,076	0.56%	1,862	6.88%	22,155.4	725.0	3.27%
**Co-morbidities**							
- Atrial Fibrillation	166,118	3.44%	3,709	2.23%	145,533.0	2,202.6	1.51%
- Asthma	677,009	14.00%	2,253	0.33%	607,561.0	1,347.8	0.22%
- Cancer	286,975	5.93%	4,489	1.56%	255,205.0	3,088.4	1.21%
- Coronary Heart Dis.	227,418	4.70%	3,666	1.61%	222,334.6	2,325.0	1.05%
- Chronic Kidney Dis.	268,605	5.55%	5,487	2.04%	258,943.8	3,154.0	1.22%

	2020 Total patients	2020 % of total	2020 Total deaths	2020 % who died	2015-9 Average total patients	2015-9 Average total deaths	2015-9 % who died
- COPD	147,546	3.05%	2,383	1.62%	134,682.6	1,569.4	1.17%
- Dementia	60,259	1.25%	4,639	7.70%	57,535.2	2,049.8	3.56%
- Diabetes	414,718	8.58%	4,435	1.07%	370,730.0	2,332.0	0.63%
- Epilepsy	72,202	1.49%	576	0.80%	68,330.8	319.2	0.47%
- Heart Failure	69,677	1.44%	2,331	3.35%	59,474.0	1,341.0	2.25%
- Hypertension	1,072,045	22.17%	9,932	0.93%	1,012,981.6	5,741.8	0.57%
- Learning Disability	22,453	0.46%	189	0.84%	19,830.6	75.4	0.38%
- Mental Health	61,017	1.26%	528	0.87%	54,660.2	255.2	0.47%
- Osteoarthritis	139,165	2.88%	2,569	1.85%	124,824.0	1,306.8	1.05%
- Rheumatoid Arthritis	47,571	0.98%	449	0.94%	42,962.8	254.8	0.59%
- Stroke or TIA	136,077	2.81%	3,056	2.25%	127,865.0	1,774.8	1.39%
