# Supplementary material for: Risk factors for excess all-cause mortality during the first wave of the COVID-19 pandemic in England: A retrospective cohort study of primary care data
Source: PLoS One. 2021 Dec 9;16(12):e0260381. doi: 10.1371/journal.pone.0260381 (PMC8659693; doi:10.1371/journal.pone.0260381)
Supplement: S2 Table — (PDF) [file pone.0260381.s005.pdf]

S2 Table: Mortality ratios for 2020 and 2015-9 (Usual) with corresponding excess mortality ratio (EMR) and true pandemic interaction (TPI) from mutually adjusted models†

	2020 Mortality Ratio (95% CI)	2015-9 Usual Mortality Ratio (UMR) (95%CI)	2020 Excess Mortality Ratio (EMR) (95%CI)	True Pandemic Interaction* (95%CI)
**Sex**				
- Females	1	1	1	1
- Males	1.407 (1.365,1.451)	1.375 (1.350,1.399)	1.470 (1.340,1.613)	1.070 (0.968,1.182)
**Age**				
- 30 to 39	0.015 (0.013,0.018)	0.018 (0.017,0.020)	0.008 (0.003,0.017)	0.418 (0.185,0.945)
- 40 to 49	0.046 (0.042,0.052)	0.054 (0.051,0.057)	0.029 (0.018,0.046)	0.533 (0.326,0.871)
- 50 to 59	0.125 (0.116,0.134)	0.131 (0.126,0.137)	0.111 (0.085,0.145)	0.848 (0.639,1.126)
- 60 to 69	0.349 (0.329,0.369)	0.367 (0.356,0.379)	0.308 (0.250,0.379)	0.839 (0.672,1.047)
- 70 to 79	1	1	1	1
- 80 to 89	3.519 (3.378,3.665)	3.189 (3.115,3.265)	4.252 (3.743,4.831)	1.334 (1.162,1.530)
- 90 to 104	9.419 (8.995,9.864)	8.002 (7.786,8.224)	12.571 (10.982,14.390)	1.571 (1.357,1.819)
**Smoking**				
- Never	1	1	1	1
- Ex	1.320 (1.276,1.365)	1.344 (1.318,1.372)	1.279 (1.163,1.407)	0.951 (0.858,1.055)
- Current	1.452 (1.379,1.528)	1.926 (1.874,1.979)	0.679 (0.555,0.831)	0.353 (0.285,0.436)
**Ethnicity**				
- White	1	1	1	1
- Black	1.322 (1.189,1.470)	0.843 (0.778,0.915)	2.201 (1.729,2.803)	2.610 (1.970,3.457)
- Asian	1.052 (0.960,1.153)	0.820 (0.768,0.874)	1.478 (1.171,1.865)	1.803 (1.386,2.345)
- Mixed	1.216 (1.049,1.410)	0.889 (0.798,0.992)	1.816 (1.262,2.615)	2.042 (1.345,3.101)
- Other	1.071 (0.936,1.225)	0.824 (0.747,0.908)	1.524 (1.085,2.142)	1.851 (1.257,2.727)
**BMI**				
- <20	2.597 (2.476,2.724)	2.584 (2.514,2.656)	2.621 (2.259,3.041)	1.014 (0.864,1.190)
- 20-30	1	1	1	1
- 30-35	0.980 (0.934,1.029)	0.938 (0.912,0.965)	1.062 (0.918,1.229)	1.132 (0.967,1.326)
- 35-40	1.201 (1.115,1.295)	1.153 (1.102,1.206)	1.296 (1.032,1.628)	1.124 (0.878,1.440)
- 40+	2.076 (1.897,2.270)	1.795 (1.695,1.900)	2.624 (2.041,3.373)	1.462 (1.108,1.929)
**Deprivation**				
- IMD1 (Least)	1	1	1	1
- IMD2	1.155 (1.102,1.209)	1.110 (1.081,1.140)	1.245 (1.078,1.437)	1.121 (0.960,1.309)
- IMD3	1.227 (1.170,1.285)	1.190 (1.159,1.222)	1.300 (1.123,1.506)	1.093 (0.933,1.279)
- IMD4	1.360 (1.296,1.426)	1.317 (1.281,1.353)	1.447 (1.246,1.680)	1.099 (0.936,1.291)
- IMD5 (Most)	1.651 (1.573,1.733)	1.569 (1.526,1.613)	1.818 (1.568,2.108)	1.159 (0.988,1.359)
**Region**				
- London vs. Rest	1.220 (1.167,1.276)	0.943 (0.917,0.971)	1.785 (1.589,2.006)	1.893 (1.664,2.152)

* - Defined as the ratio of the EMR to the UMR.
† - All models include terms for all factors listed in above table.
