# Supplementary material for: Risk factors for excess all-cause mortality during the first wave of the COVID-19 pandemic in England: A retrospective cohort study of primary care data
Source: PLoS One. 2021 Dec 9;16(12):e0260381. doi: 10.1371/journal.pone.0260381 (PMC8659693; doi:10.1371/journal.pone.0260381)
Supplement: S3 Table — (PDF) [file pone.0260381.s006.pdf]

S3 Table: Mortality ratios for 2020 and 2015-9 (Usual) with corresponding excess mortality ratio (EMR) and true pandemic interaction (TPI) for sex, smoking, ethnicity, deprivation, BMI and region stratified by age

	2020 Mortality Ratio (95% CI)	2015-9 Usual Mortality Ratio (UMR) (95%CI)	2020 Excess Mortality Ratio (EMR) (95%CI)	True Pandemic Interaction* (95%CI)
**Sex (Male vs Female)**				
- 30 to 64	1.430 (1.322,1.546)	1.407 (1.349,1.468)	1.492 (1.097,2.029)	1.060 (0.766,1.468)
- 65 to 79	1.567 (1.474,1.666)	1.503 (1.453,1.555)	1.727 (1.388,2.150)	1.149 (0.910,1.451)
- 80+	1.348 (1.296,1.403)	1.276 (1.246,1.306)	1.479 (1.321,1.655)	1.159 (1.026,1.310)
**Smoking (Ex vs Never)**				
- 30 to 64	1.556 (1.418,1.706)	1.530 (1.451,1.614)	1.603 (1.210,2.124)	1.048 (0.773,1.420)
- 65 to 79	1.468 (1.367,1.576)	1.603 (1.538,1.671)	1.236 (0.989,1.545)	0.771 (0.606,0.981)
- 80+	1.193 (1.144,1.244)	1.145 (1.116,1.174)	1.275 (1.136,1.432)	1.114 (0.982,1.264)
**Smoking (Current vs Never)**				
- 30 to 64	2.193 (1.982,2.426)	2.969 (2.815,3.131)	0.716 (0.429,1.197)	0.241 (0.141,0.412)
- 60 to 79	2.153 (1.963,2.361)	2.724 (2.591,2.865)	1.176 (0.826,1.675)	0.432 (0.297,0.628)
- 80+	1.153 (1.053,1.264)	1.397 (1.331,1.467)	0.741 (0.537,1.023)	0.531 (0.377,0.747)
**Ethnicity (Black vs White)**				
- 30 to 64	1.703 (1.420,2.042)	1.028 (0.902,1.171)	3.622 (2.212,5.930)	3.525 (2.018,6.156)
- 60 to 79	1.906 (1.550,2.343)	0.962 (0.821,1.126)	4.045 (2.536,6.452)	4.206 (2.438,7.257)
- 80+	1.149 (0.975,1.354)	0.762 (0.668,0.870)	1.754 (1.224,2.513)	2.301 (1.501,3.526)
**Ethnicity (Asian vs White)**				
- 30 to 64	1.220 (1.034,1.439)	0.671 (0.593,0.759)	2.779 (1.813,4.260)	4.140 (2.539,6.751)
- 60 to 79	1.071 (0.900,1.275)	0.864 (0.772,0.967)	1.541 (0.929,2.555)	1.784 (1.021,3.117)
- 80+	0.950 (0.824,1.094)	0.836 (0.757,0.923)	1.128 (0.789,1.612)	1.350 (0.900,2.024)
**BMI (<20 vs 20-30)**				
- 30 to 64	2.920 (2.533,3.367)	3.345 (3.112,3.595)	1.614 (0.709,3.674)	0.483 (0.206,1.131)
- 65 to 79	3.410 (3.065,3.795)	3.853 (3.637,4.081)	2.322 (1.440,3.742)	0.603 (0.365,0.995)
- 80+	2.431 (2.295,2.575)	2.324 (2.245,2.405)	2.607 (2.232,3.047)	1.122 (0.947,1.330)
**BMI (30-35 vs 20-30)**				
- 30 to 64	1.137 (1.017,1.271)	0.983 (0.923,1.047)	1.611 (1.072,2.422)	1.639 (1.061,2.533)
- 65 to 79	1.098 (1.008,1.196)	0.991 (0.944,1.041)	1.360 (1.024,1.807)	1.372 (1.011,1.862)
- 80+	0.941 (0.878,1.008)	0.939 (0.901,0.979)	0.943 (0.775,1.146)	1.004 (0.811,1.242)
**BMI (35-40 vs 20-30)**				
- 30 to 64	1.568 (1.351,1.821)	1.208 (1.104,1.322)	2.677 (1.638,4.377)	2.216 (1.302,3.773)
- 65 to 79	1.346 (1.186,1.526)	1.305 (1.214,1.402)	1.446 (0.919,2.274)	1.108 (0.683,1.798)
- 80+	1.132 (1.003,1.279)	1.127 (1.046,1.214)	1.141 (0.810,1.609)	1.013 (0.695,1.476)
**BMI (40- vs 20-30)**				
- 30 to 64	3.187 (2.753,3.689)	2.184 (1.987,2.401)	6.274 (4.032,9.761)	2.873 (1.766,4.671)
- 65 to 79	2.406 (2.081,2.781)	1.929 (1.760,2.114)	3.580 (2.312,5.544)	1.856 (1.150,2.997)
- 80+	1.449 (1.186,1.769)	1.605 (1.400,1.840)	1.190 (0.638,2.221)	0.741 (0.377,1.456)
**IMD (2 vs 1 - Least)**				
- 30 to 64	1.267 (1.109,1.448)	1.169 (1.088,1.255)	1.599 (0.917,2.790)	1.369 (0.761,2.460)
- 65 to 79	1.205 (1.095,1.326)	1.146 (1.086,1.208)	1.364 (0.956,1.945)	1.190 (0.816,1.737)
- 80+	1.154 (1.090,1.223)	1.115 (1.078,1.152)	1.228 (1.036,1.457)	1.102 (0.916,1.326)
**IMD (3 vs 1 - Least)**				
- 30 to 64	1.465 (1.285,1.670)	1.389 (1.296,1.488)	1.722 (0.976,3.038)	1.240 (0.683,2.252)
- 65 to 79	1.387 (1.260,1.526)	1.338 (1.270,1.411)	1.515 (1.055,2.175)	1.132 (0.771,1.662)
- 80+	1.192 (1.124,1.264)	1.138 (1.099,1.177)	1.293 (1.088,1.537)	1.137 (0.943,1.371)
**IMD (4 vs 1 - Least)**				

	2020 Mortality Ratio (95% CI)	2015-9 Usual Mortality Ratio (UMR) (95%CI)	2020 Excess Mortality Ratio (EMR) (95%CI)	True Pandemic Interaction* (95%CI)
- 30 to 64	1.838 (1.619,2.087)	1.704 (1.592,1.823)	2.291 (1.344,3.904)	1.345 (0.767,2.357)
- 65 to 79	1.704 (1.548,1.876)	1.625 (1.541,1.714)	1.915 (1.340,2.735)	1.178 (0.806,1.722)
- 80+	1.258 (1.183,1.338)	1.191 (1.149,1.235)	1.383 (1.155,1.655)	1.161 (0.955,1.411)
**IMD (5 vs 1 - Least)**				
- 30 to 64	2.485 (2.199,2.808)	2.335 (2.189,2.492)	2.987 (1.770,5.041)	1.279 (0.737,2.218)
- 65 to 79	2.355 (2.142,2.588)	2.167 (2.056,2.283)	2.857 (2.040,4.002)	1.319 (0.921,1.889)
- 80+	1.457 (1.366,1.555)	1.322 (1.273,1.373)	1.710 (1.425,2.052)	1.294 (1.061,1.578)
**Region (London vs Non)**				
- 30 to 64	1.424 (1.308,1.550)	1.037 (0.983,1.094)	2.454 (1.914,3.145)	2.367 (1.802,3.109)
- 65 to 79	1.220 (1.167,1.276)	0.943 (0.917,0.971)	1.785 (1.589,2.006)	1.893 (1.664,2.152)
- 80+	1.175 (1.122,1.230)	1.130 (1.101,1.160)	1.271 (1.094,1.475)	1.124 (0.958,1.321)

* - Defined as the ratio of the EMR to the UMR (see S1 Appendix). Note that all models adjust for age and sex.
