# Supplementary material for: Risk factors for excess all-cause mortality during the first wave of the COVID-19 pandemic in England: A retrospective cohort study of primary care data
Source: PLoS One. 2021 Dec 9;16(12):e0260381. doi: 10.1371/journal.pone.0260381 (PMC8659693; doi:10.1371/journal.pone.0260381)
Supplement: S4 Table — (PDF) [file pone.0260381.s007.pdf]

S4 Table: Mortality ratios for 2020 and 2015-9 (Usual) with corresponding excess mortality ratio (EMR) and true pandemic interaction (TPI) for selected co-morbidities stratified by age

	2020 Mortality Ratio (95% CI)	2015-9 Usual Mortality Ratio (UMR) (95%CI)	2020 Excess Mortality Ratio (EMR) (95%CI)	True Pandemic Interaction* (95%CI)
**Asthma**				
- 30 to 64	1.248 (1.124,1.386)	1.313 (1.240,1.390)	1.082 (0.700,1.672)	0.824 (0.521,1.305)
- 65 to 79	1.187 (1.089,1.294)	1.267 (1.208,1.329)	1.007 (0.727,1.393)	0.794 (0.562,1.122)
- 80+	1.065 (1.003,1.131)	1.058 (1.021,1.097)	1.076 (0.908,1.276)	1.017 (0.844,1.225)
**Cancer**				
- 30 to 64	6.795 (6.212,7.432)	9.013 (8.565,9.485)	2.218 (1.398,3.518)	0.246 (0.152,0.397)
- 65 to 79	3.078 (2.887,3.282)	4.158 (4.015,4.306)	1.198 (0.901,1.595)	0.288 (0.213,0.389)
- 80+	1.367 (1.305,1.432)	1.613 (1.570,1.656)	0.991 (0.856,1.147)	0.615 (0.525,0.720)
**Coronary Heart Disease**				
- 60 to 79	1.750 (1.621,1.889)	1.741 (1.669,1.817)	1.770 (1.361,2.302)	1.016 (0.768,1.345)
- 80+	1.276 (1.219,1.335)	1.314 (1.280,1.349)	1.213 (1.065,1.381)	0.923 (0.802,1.063)
**COPD**				
- 60 to 79	2.678 (2.481,2.891)	3.246 (3.116,3.381)	1.515 (1.094,2.097)	0.467 (0.331,0.657)
- 80+	1.592 (1.500,1.690)	1.756 (1.697,1.817)	1.326 (1.105,1.591)	0.755 (0.620,0.919)
**Dementia**				
- 60 to 79	9.552 (8.782,10.389)	5.181 (4.887,5.493)	22.400 (18.069,27.769)	4.323 (3.395,5.506)
- 80+	4.061 (3.897,4.231)	2.613 (2.546,2.682)	7.847 (7.042,8.744)	3.003 (2.666,3.383)
**Diabetes**				
- 30 to 64	2.971 (2.716,3.251)	2.073 (1.961,2.192)	6.355 (4.719,8.559)	3.065 (2.223,4.227)
- 65 to 79	1.979 (1.853,2.114)	1.774 (1.707,1.843)	2.511 (2.006,3.143)	1.416 (1.113,1.801)
- 80+	1.435 (1.371,1.502)	1.328 (1.291,1.365)	1.627 (1.436,1.868)	1.226 (1.068,1.425)
**Epilepsy**				
- 30 to 64	3.961 (3.366,4.661)	3.670 (3.350,4.020)	4.743 (2.635,8.536)	1.292 (0.691,2.418)
- 65 to 79	2.718 (2.345,3.150)	2.235 (2.042,2.447)	3.863 (2.479,6.019)	1.728 (1.065,2.805)
- 80+	1.629 (1.431,1.854)	1.448 (1.337,1.568)	1.934 (1.378,2.714)	1.336 (0.919,1.941)
**Hypertension**				
- 30 to 64	1.801 (1.658,1.956)	1.414 (1.347,1.485)	3.243 (2.415,4.356)	2.294 (1.678,3.136)
- 65 to 79	1.326 (1.248,1.409)	1.133 (1.095,1.172)	1.934 (1.550,2.411)	1.707 (1.349,2.159)
- 80+	1.038 (0.995,1.082)	0.982 (0.959,1.006)	1.140 (1.010,1.286)	1.160 (1.018,1.322)
**Learning Disability**				
- 30 to 64	7.550 (6.117,9.318)	4.828 (4.181,5.574)	15.116 (8.451,27.037)	3.131 (1.634,5.999)
- 65 to 79	5.499 (4.347,6.957)	3.540 (2.984,4.200)	10.128 (5.509,18.618)	2.861 (1.430,5.724)
**Mental Health**				
- 30 to 64	4.131 (3.483,4.901)	4.058 (3.684,4.469)	4.330 (2.230,8.407)	1.067 (0.527,2.161)
- 65 to 79	3.625 (3.110,4.224)	2.757 (2.499,3.041)	5.699 (3.680,8.825)	2.067 (1.276,3.349)
- 80+	2.468 (2.156,2.824)	1.641 (1.492,1.806)	3.879 (2.863,5.255)	2.364 (1.668,3.350)

* - Defined as the ratio of the EMR to the UMR (see S1 Appendix). Note that all models adjust for age and sex.
