# Supplementary material for: Risk factors for excess all-cause mortality during the first wave of the COVID-19 pandemic in England: A retrospective cohort study of primary care data
Source: PLoS One. 2021 Dec 9;16(12):e0260381. doi: 10.1371/journal.pone.0260381 (PMC8659693; doi:10.1371/journal.pone.0260381)
Supplement: S5 Table — (PDF) [file pone.0260381.s008.pdf]

S5 Table: Mortality ratios for 2020 and 2015-9 (Usual) with corresponding excess mortality ratio (EMR) and true pandemic interaction (TPI) for selected co-morbidities stratified by care home residence

	2020 Mortality Ratio (95% CI)	2015-9 Usual Mortality Ratio (UMR) (95%CI)	2020 Excess Mortality Ratio (EMR) (95%CI)	True Pandemic Interaction* (95%CI)
**Dementia**				
- Care Home only	1.897 (1.725,2.087)	1.507 (1.409,1.612)	2.308 (1.922,2.772)	1.532 (1.240,1.892)
- No Care Home	4.652 (4.474,4.838)	2.961 (2.887,3.037)	9.716 (8.722,10.823)	3.281 (2.915,3.692)
**Learning Disability**				
- Care Home only	0.657 (0.518,0.833)	0.692 (0.576,0.831)	0.629 (0.402,0.985)	0.909 (0.537,1.539)
- No Care Home	5.150 (4.273,6.207)	3.455 (3.046,3.919)	8.954 (5.550,14.447)	2.592 (1.514,4.435)
**Mental Health**				
- Care Home only	0.806 (0.670,0.971)	0.744 (0.646,0.856)	0.857 (0.612,1.199)	1.152 (0.774,1.714)
- No Care Home	3.011 (2.726,3.325)	2.480 (2.333,2.636)	4.214 (3.149,5.638)	1.699 (1.236,2.337)

* - Defined as the ratio of the EMR to the UMR (see S1 Appendix). Note that all models adjust for age and sex.
