# Supplementary material for: Risk factors for excess all-cause mortality during the first wave of the COVID-19 pandemic in England: A retrospective cohort study of primary care data
Source: PLoS One. 2021 Dec 9;16(12):e0260381. doi: 10.1371/journal.pone.0260381 (PMC8659693; doi:10.1371/journal.pone.0260381)
Supplement: S6 Table — (PDF) [file pone.0260381.s009.pdf]

S6 Table: Mortality ratios for 2020 and 2015-9 (Usual) with corresponding excess mortality ratio (EMR) and true pandemic interaction (TPI) for haematological and non- haematological cancer

	2020 Mortality Ratio (95% CI)	2015-9 Usual Mortality Ratio (UMR) (95%CI)	2020 Excess Mortality Ratio (EMR) (95%CI)	True Pandemic Interaction* (95%CI)
**Cancer**				
- Haematological	2.113 (1.950,2.291)	2.390 (2.285,2.501)	1.580 (1.181,2.114)	0.661 (0.485,0.901)
- Non-haematological	1.966 (1.896,2.038)	2.501 (2.451,2.552)	1.064 (0.929,1.219)	0.426 (0.369,0.491)

* - Defined as the ratio of the EMR to the UMR (see S1 Appendix). Note that all models adjust for age and sex.
