# Supplementary material for: Risk factors for excess all-cause mortality during the first wave of the COVID-19 pandemic in England: A retrospective cohort study of primary care data
Source: PLoS One. 2021 Dec 9;16(12):e0260381. doi: 10.1371/journal.pone.0260381 (PMC8659693; doi:10.1371/journal.pone.0260381)
Supplement: Attachment — Submitted filename: Response to Reviewers R2.docx [file pone.0260381.s011.docx]

6. Review Comments to the Author

Please use the space provided to explain your answers to the questions above. You may also include additional comments for the author, including concerns about dual publication, research ethics, or publication ethics. (Please upload your review as an attachment if it exceeds 20,000 characters)

Reviewer #3: Previous comments have been adequately addressed.

The Authors may wish to re-revise changes to the two sentences from lines 291-296 beginning "By analysing individual risk factors...", as their meaning is currently unclear

**We apologise for the minor punctuation error where a full stop had not been marked for deletion.**

**The corrected sentence now reads**

**“By analysing individual risk factors for excess mortality, and also by presenting the excess mortality ratios in the context of the usual mortality ratios, we think the methodology we have developed for studying the impact of individual risk factors on excess mortality and identifying the true pandemic effect (interaction) for each risk factor provides a template that can be generalised across the COVID-19 pandemic, as well as to other causes of excess mortality such as influenza, and will be less susceptible to ecologic bias”**
